# Ingested insecticide to control *Aedes aegypti*: developing a novel dried attractive toxic sugar bait device for intra-domiciliary control

**DOI:** 10.1186/s13071-020-3930-9

**Published:** 2020-02-17

**Authors:** Rachel Sippy, Galo E. Rivera, Valeria Sanchez, Froilán Heras, Bianca Morejón, Efraín Beltrán, Robert S. Hikida, María A. López-Latorre, Alex Aguirre, Anna M. Stewart-Ibarra, David A. Larsen, Marco Neira

**Affiliations:** 1 Institute for Global Health & Translational Science, SUNY-Upstate Medical University, Syracuse, NY USA; 2 Department of Geography, University of Florida, Gainesville, FL USA; 3 Center for Research on Health in Latin America, Pontificia Universidad Católica del Ecuador, Quito, Ecuador; 4 Present Address: Vector Biology Group, Max Plank Institute for Infection Biology, Berlin, Germany; 5 Unidad Académica de Ciencias Químicas y de la Salud, Universidad Técnica de Machala, Machala, Ecuador; 6 Present Address: Biology Division, College of Arts and Sciences, Kansas State University, Manhattan, KS USA; 7 Department of Medicine, SUNY-Upstate Medical University, Syracuse, NY USA; 8 Department of Public Health and Preventative Medicine, SUNY-Upstate Medical University, Syracuse, NY USA; 9 Present Address: Medical School, College of Health Sciences, Universidad de las Américas, Quito, Ecuador; 10 Present Address: InterAmerican Institute for Global Change Research (IAI), Montevideo, Uruguay; 11 Department of Public Health, Syracuse University, Syracuse, NY USA; 12 Ohio University, Athens, Ohio USA

**Keywords:** *Aedes aegypti*, Vector control, Toxic sugar bait, Attractive bait, Semi-field, Dengue, Arbovirus, ATSB

## Abstract

**Background:**

Illnesses transmitted by *Aedes aegypti* (Linnaeus, 1762) such as dengue, chikungunya and Zika comprise a considerable global burden; mosquito control is the primary public health tool to reduce disease transmission. Current interventions are inadequate and insecticide resistance threatens the effectiveness of these options. Dried attractive bait stations (DABS) are a novel mechanism to deliver insecticide to *Ae. aegypti*. The DABS are a high-contrast 28 inch^2^ surface coated with dried sugar-boric acid solution. *Aedes aegypti* are attracted to DABS by visual cues only, and the dried sugar solution elicits an ingestion response from *Ae. aegypti* landing on the surface. The study presents the development of the DABS and tests of their impact on *Ae. aegypti* mortality in the laboratory and a series of semi-field trials.

**Methods:**

We conducted multiple series of laboratory and semi-field trials to assess the survivability of *Ae. aegypti* mosquitoes exposed to the DABS. In the laboratory experiments, we assessed the lethality, the killing mechanism, and the shelf life of the device through controlled experiments. In the semi-field trials, we released laboratory-reared female *Ae. aegypti* into experimental houses typical of peri-urban tropical communities in South America in three trial series with six replicates each. Laboratory experiments were conducted in Quito, Ecuador, and semi-field experiments were conducted in Machala, Ecuador, an area with abundant wild populations of *Ae. aegypti* and endemic arboviral transmission.

**Results:**

In the laboratory, complete lethality was observed after 48 hours regardless of physiological status of the mosquito. The killing mechanism was determined to be through ingestion, as the boric acid disrupted the gut of the mosquito. In experimental houses, total mosquito mortality was greater in the treatment house for all series of experiments (*P* < 0.0001).

**Conclusions:**

The DABS devices were effective at killing female *Ae. aegypti* under a variety of laboratory and semi-field conditions. DABS are a promising intervention for interdomiciliary control of *Ae. aegypti* and arboviral disease prevention.
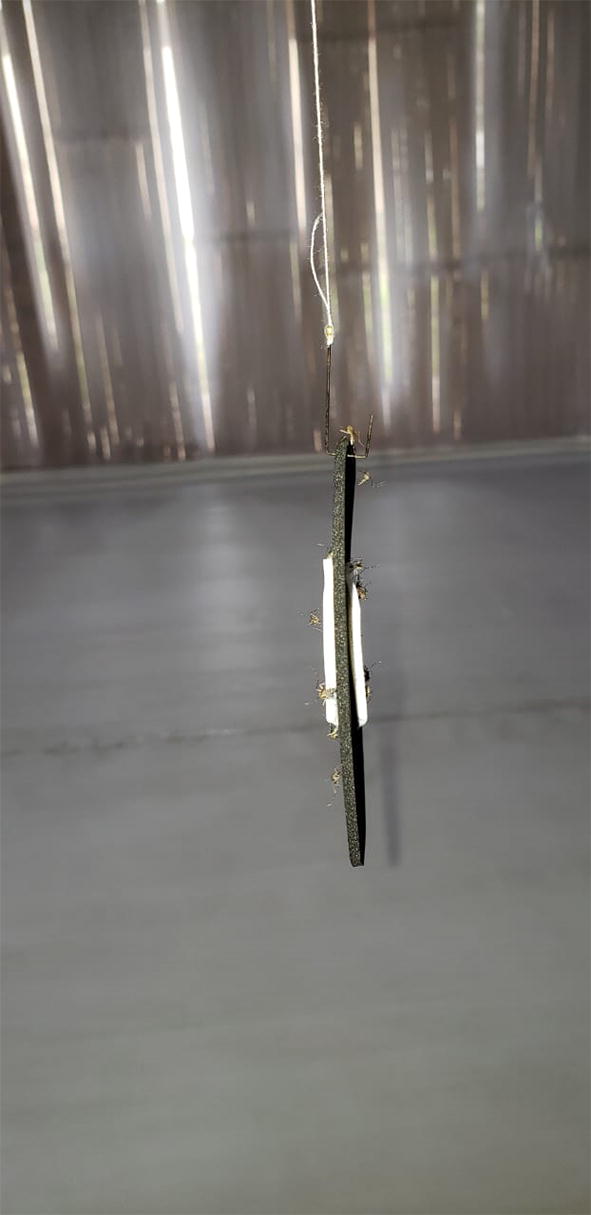

## Background

Arboviral illnesses, including dengue, chikungunya, yellow fever and Zika, are major contributors to morbidity and mortality in the tropics and subtropics. The burden is particularly apparent in Central and South America; between 2010–2018, the estimated annual number of dengue cases in the region ranged from 500,000 to 2,400,000 [1], and since 2013 Pan American Health Organization has estimated that there have been more than 2.5 million suspected and confirmed cases of chikungunya and 800,000 cases of Zika. The viruses causing these diseases are spread mainly by the mosquitoes *Aedes aegypti* (Linnaeus, 1762) and *Aedes albopictus* (Skuse, 1894), with *Ae. aegypti* serving as the principal vector in many South American countries, including Ecuador [2]. Due to the lack of commercially available vaccines for most human arboviral diseases, prevention efforts focus on vector surveillance and control methods [3].

Vector control relies heavily on contact-based insecticides, which are available in four main classes: organophosphates, pyrethroids, carbamates and organochlorines. Indoor residual spraying is a common approach to vector control, for which twelve insecticides are available and approved for human use [4]. This small number of approved insecticides constitutes an impediment for the implementation of effective vector control strategies (such as pesticide rotation cycles) aimed at decreasing the development of resistance to any single insecticide [5]. As a result, pesticide resistance has become a major limitation for current vector control strategies, and is widespread in South American countries [6–8]. Our current reliance on a few chemical molecules to control *Ae. aegypti* is an increasingly flawed strategy, as evidenced by the proliferation of this disease vector across the globe and increasing arbovirus epidemics [9].

In contrast to the contact-based insecticide approach of the public health sector, the agricultural industry has focused on ingested insecticides for pest control. The use of ingested insecticides could be applied in disease control programmes and interventions if disease vectors are successfully led to ingest the insecticide. One solution, attractive toxic sugar baits (ATSB), exploits the nectar-feeding behavior of mosquitoes [10, 11] to deliver the insecticide. An ATSB uses a mixture of a lethal agent with sugar water and an additional attractant [12]. ATSBs have been tested for *Anopheles* spp. [13–17], *Culex* spp. [15, 16, 18, 19], *Ae. albopictus* [20–23], and other vector or nuisance species [16] with a variety of attractants, baits, active ingredients, designs, and placement strategies. Although laboratory bioassays demonstrate that ATSBs are toxic to *Ae. aegypti* [16, 24, 25], semi-field and field evaluations have had poor results in reducing *Ae. aegypti* populations [26, 27], indicating that ATSB devices must be carefully designed and tested for each target species [12].

Compared to other mosquito species, *Ae. aegypti* appear to have a lower propensity for sugar-feeding, preferring human blood meals instead [11]. Despite this, *Ae. aegypti* females will readily feed on sugar in the laboratory, and often feed on plant sugars in the wild [28–31]. However, traditional attractive sugar bait strategies that rely only on fruit volatiles as an attractant are likely insufficient to “lure” highly anthropophilic female *Ae. aegypti* in the natural environment.

**Fig. 1:**
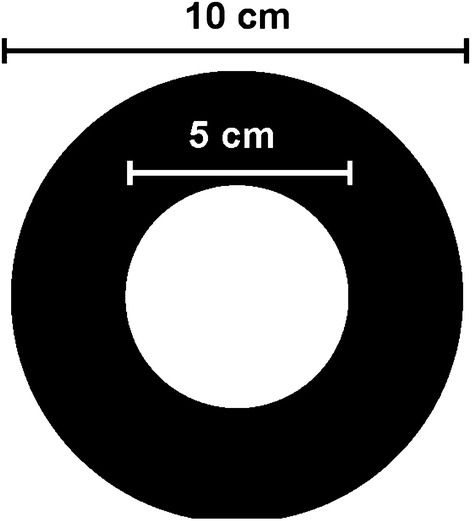
Dry attractive bait stations (DABS)

Herein we present the development of dried attractive bait stations (DABS) (Fig. 1), and show results from laboratory and semi-field experiments. In the laboratory we first identified the lethality of DABS (Series 1.1), aimed to identify the killing mechanism of the DABS (Series 1.2), assessed how the physiological status altered the effectiveness of DABS (Series 1.3), and assessed the shelf life of the DABS (Series 1.4). In the semi-field trials, we sought to determine the timing of mosquito mortality (Series 2.1), assess the relationship between DABS exposure time and mosquito mortality (Series 2.2), and to demonstrate these effects in the presence of competing attractants (Series 2.3).

## Methods

### Study setting

#### Laboratory experiments

Laboratory experiments were conducted at the Center for Research on Health in Latin America (CISeAL, by its Spanish acronym), where they were reared and maintained under standard insectary conditions: 28 ± 1 °C temperature, 80 ± 10% relative humidity, and a 12 h:12 h (L:D) photocycle. Larvae were fed finely ground fish food. When required, mosquitoes were sexed during the pupal stage. Adults were kept in 20 × 20 × 20 cm cages. For maintenance, adult mosquitoes were fed 10% sucrose solution *ad libitum*. For blood-feeding, female adult mosquitoes were offered access to a restrained female mouse. All mosquitoes were maintained under insectary conditions after adult emergence before they were used for experiments. Mosquitoes referred to as “starved” hereafter were deprived of access to sugar or blood (but not water) for 48 h prior to their use in experiments.

#### Semi-field trials

Trials were performed in experimental houses meant to emulate typical housing found in areas with active dengue transmission. Photographs of the houses are available in Additional file 1: Figure S1. The houses are constructed of wood and cane and are raised on a 1-m platform with walkways to improve structural integrity and facilitate window access; one window on each house is equipped with window escape traps with sleeves to monitor escape behavior. The dimensions of the houses are 3.85 m wide × 4.85 m long × 3 m high. Each house has three windows (0.9 m wide × 0.6 m high) and one door (1.03 m wide × 3 m high). The house frames are made of wood; they have untreated wooden plank flooring, walls of untreated cane, and a roof of zinc panels. The window traps are 0.45 m long × 0.66 m wide × 0.45 m high. The houses are located on campus at the Universidad Técnica de Machala in the city of Machala, Ecuador (3°15′S, 79°57′W), a region with abundant wild populations of *Ae. aegypti* and endemic arbovirus transmission. Experiments were conducted under ambient climate conditions (temperature range: 23.1–35.6 °C, mean temperature: 28.4 °C, relative humidity range: 43.9–95.0%, mean relative humidity: 75.8%). Each trial replicate was conducted with one control and one experimental house; the specific house used as the experimental or control house was alternated upon each replicate.

### Biological material

*Aedes aegypti* eggs were provided by the Center for Research on Health in Latin America (CISeAL, by its Spanish acronym) at the Pontificia Universidad Católica del Ecuador. All strains used in this study originated from Ecuador, and had been maintained in laboratory conditions since 2015. The laboratory experiments were performed with strains originally collected in Ecuador from the cities of Guayaquil and Puerto Francisco de Orellana. The semi-filed study was performed with a strain originally collected in the city of Machala.

#### Semi-field experiments

Hatching and rearing of *Ae. aegypti* for the semi-field experiments were performed at the Laboratory of Entomology at the Universidad Técnica de Machala. Considering this laboratory is located in a region where *Ae. aegypti* actively reproduces and thrives, environmental conditions (temperature: 28–32 °C; relative humidity: 60–80%) were not artificially controlled in the mosquito-rearing facilities. A vacuum pressure system was used to synchronize egg hatching (one-hour exposure to obtain first-stage larvae). Larvae were fed with finely ground fish food. At the pupal stage, males and females were separated. Adults were kept in 20 × 20 × 20 cm cages. Adults were fed on 10% sugar solution *ad libitum*. Each experimental semi-field experiment series used nulliparous females aged 1–5 days and starved for 24 h prior to experimental release.

### Dried attractive bait stations (DABS)

The DABS device consists of two concentric foam disks (an inner white disk 1 cm in diameter, and an outer black disk 8 cm in diameter). Experimental DABS were impregnated with a 10% sucrose solution containing 1% boric acid as a lethal agent. Control DABS were impregnated with 10% sucrose solution without boric acid (US Patent Application 15/990,931, 2018).

### Laboratory experiments

#### Series 1.1: Survival assessment of mosquitoes exposed to the device

To determine whether exposure to the DABS devices has an influence on adult mosquito survival probability, we conducted an experiment in which groups of 30 adult female mosquitoes, placed in a 15 × 15 × 15 cm cage, were exposed during 48 h to either a DABS device or a control device (sugar solution but no boric acid). We replicated each experiment four times. The assessment was repeated using each of the two laboratory strains described previously.

#### Series 1.2: Appraisal of biological mode of action of the device

To establish whether the toxic component of DABS needs to be ingested by the mosquitoes in order to exert its effect, we presented the devices to cohorts of adult females aged 1–7 days, which were unable to ingest food due to the surgical ablation of their mouthparts. To establish these cohorts, individual mosquitoes were first anesthetized by placing them at 4 °C for 10–15 min. Anesthetized specimens were individually placed under a dissection microscope and, using a human hair, we tied a knot at the proboscis’ proximal end in order to create a constriction that would impede the flow of food. Subsequently, the part of the proboscis anterior to the knot was removed using micro-dissection scissor. Following the surgery, mosquitoes were left to rest for 24 h before being used in any experiment. To control for the potential negative effect of the anesthetizing procedure on mosquito survival, non-ablated mosquitoes used in the control groups were also placed at 4 °C for 10–15 min, and allowed to recover for 24 h before experimental set-up.

We conducted the experiment with four separate cages, each with 20 starved mosquitoes. We treated cage 1 with toxic DABS devices and used 20 ablated mosquitoes; cage 2 held non-toxic control devices and 20 ablated mosquitoes. We treated cage 3 with toxic DABS devices and non-ablated mosquitoes; cage 4 held a non-toxic control device and non-ablated mosquitoes. We assessed mortality in all groups at 24 and 48 h of exposure to the devices. We replicated the experiment three times.

We then conducted an experiment wherein 30 adult starved female mosquitoes aged 1–7 days were introduced to a cage with a DABS device, and 30 adult starved female mosquitoes of similar age were introduced to a cage with a non-toxic control device. We monitored cages for 24 h and removed dead mosquitoes by aspiration every hour from the cages. Using a dissection microscope, we removed the legs, head and wings of every dead specimen and placed onto a drop of 70% ethanol. Through this process we gently disrupted the abdominal cuticle to permit the exposure of internal tissues to the fixative. Afterwards we fixed individual mosquitoes in a solution containing 2.5% glutaraldehyde, 2.5% paraformaldehyde in 0.1 M cacodylate buffer (pH 7.4), and stored them at 4 °C for 72 h. We then washed specimens in cacodylate buffer with 0.1 M sucrose overnight. Post-fixing was achieved by leaving the specimens for 2 h at 4 °C in 2% osmium tetroxide in 0.1 cacodylate buffer (pH 7.4). Subsequently, individuals were stained using 2% uranyl acetate and left to rest for 3 h in the dark at room temperature. Tissues were later dehydrated through a series of ethanol baths (50%, 70%, 95%, 100%). Afterwards, they were placed in propylene oxide for 30 min, then in a 1:1 volume propylene oxide resin mixture (Epon 812, Araldite 502, dodecenyl succinic anhydride, benzyl dimethylamine) for 1 h and later, one more volume of resin was added and left on a rotator overnight. Finally, mosquitoes were embedded in resin and incubated at 60 °C for 24 h. Resin samples were stained using 2% uranyl acetate. We then utilized a transmission electron microscope to observe specimens and obtain micrographs of relevant tissues.

#### Series 1.3: Effects of the physiological status of the mosquitoes on the performance of DABS

We examined two different physiological statuses using mated starved female adult mosquitoes aged 1–7 days, namely blood-fed and parous. We established females deemed as “blood-fed” by selecting blood-engorged individuals immediately after a blood meal. We established females deemed as “parous” by first blood-feeding and subsequently maintaining mosquitoes for 7 days under insectary conditions in order to ensure that they had oviposited before being used for experimentation. We set up two cages for each of the defined physiological statuses, each with 30 mosquitoes. One cage exposed the mosquitoes to an ATSB device, and the other held a control non-toxic device. We gathered survival data at 24 and 48 h following introduction to the cages, and replicated these experiments three times.

#### Series 1.4: Shelf-life of the device

In order to determine the shelf life of ATSB devices, toxicity tests were performed using devices which had been stored for 38, 80 and 118 days after their production. For storage, devices were individually wrapped inside a sealed plastic bag and placed in an incubator at 28 ± 2 °C and 80 ± 10% relative humidity. We conducted three replicates of previously described experiments for each storage time.

### Semi-field trials

#### Series 2.1: 24 hours of DABS exposure in experimental houses

Each house contained four DABS devices (control or treatment DABS as appropriate) suspended on strings attached to the roof of the house at a height of 30–50 cm above ground and approximately 30 cm from the nearest wall. For each trial replicate, 50 female *Ae. aegypti* were released into each house through the escape window sleeve (release time 11:00–14:00 h). Twenty-four hours after release, dead mosquitoes were collected from the floor and window escape traps in each house, and the remaining live mosquitoes were captured with a hand-held aspirator (Prokopack, John W. Hock Company, Gainesville, USA). All live mosquitoes were labeled by experimental group and observed for 48 additional hours in laboratory cages (under laboratory conditions with food available). Mortality was calculated for 24 h, 48 h and 72 h. Six trial replicates were performed for Series 1.

#### Series 2.2: 48 hours of DABS exposure in experimental houses

Each house contained four DABS devices (control or treatment DABS as appropriate) and two sources of water (wet cotton in a black plastic bucket). For each trial replicate, 50 female *Ae. aegypti* were released into each house through the escape window sleeve (release time 8:00–11:00 h). Forty-eight hours after release, dead mosquitoes were collected in each house and remaining live mosquitoes were captured with an aspirator. Mortality was calculated for 48 h. Six replicates were performed for Series 2.

#### Series 2.3: 48 hours of DABS exposure in experimental houses with competing attractant

Each house contained four DABS devices (control or treatment DABS as appropriate), two sources of water (wet cotton in a black plastic bucket), and 100 g of peeled, cut apples in a dish placed on a chair in the center of the house as a competing attractant. Recently emerged female *Ae. aegypti* rely on sugar meals for energy; these meals may include aging fruit and female *Ae. aegypti* will feed on fructose (as is found in apples). For each trial replicate, 50 female *Ae. aegypti* were released into each house through the escape window sleeve (release time 9:00–12:00 h). Forty-eight hours after release, dead mosquitoes were collected in each house and remaining live mosquitoes were captured with an aspirator. Mortality was calculated for 48 h. Six replicates were performed for Series 3.

### Statistical analyses

For the Series 1 experiments, data was processed, plotted, and analyzed using Python v2.7.13. For data processing we used the Pandas v0.22.0 module. Plots were generated using the Plotly v3.10.0 module. We examined the normal distribution of the data with Kolmogorov–Smirnov and Shapiro–Wilk tests. In experiments in Series 1.1, 1.3 and 1.4 Student’s t-test comparisons were performed using the Scipy v1.0.0 module. In Series 1.2, one-way ANOVA was performed using the Scipy v1.0.0 module with four experimental groups. Tukey’s range test, using Statsmodels v.0.10.0 module, was performed after ANOVA for determining ranges for each group. All data and codes used for the data have been stored in a private online git repository and are provided upon request. In Series 2.1–2.3, mosquito mortality data from each series were compared using a two-tailed paired t-test (paired by replicate). Mean mosquito mortality was compared across series using a two-tailed t-test. Data were analyzed using Excel (Microsoft, Redmond, USA).

## Results

### Laboratory experiments

#### Series 1.1: Effects of DABS exposure on mosquito survival

**Fig. 2:**
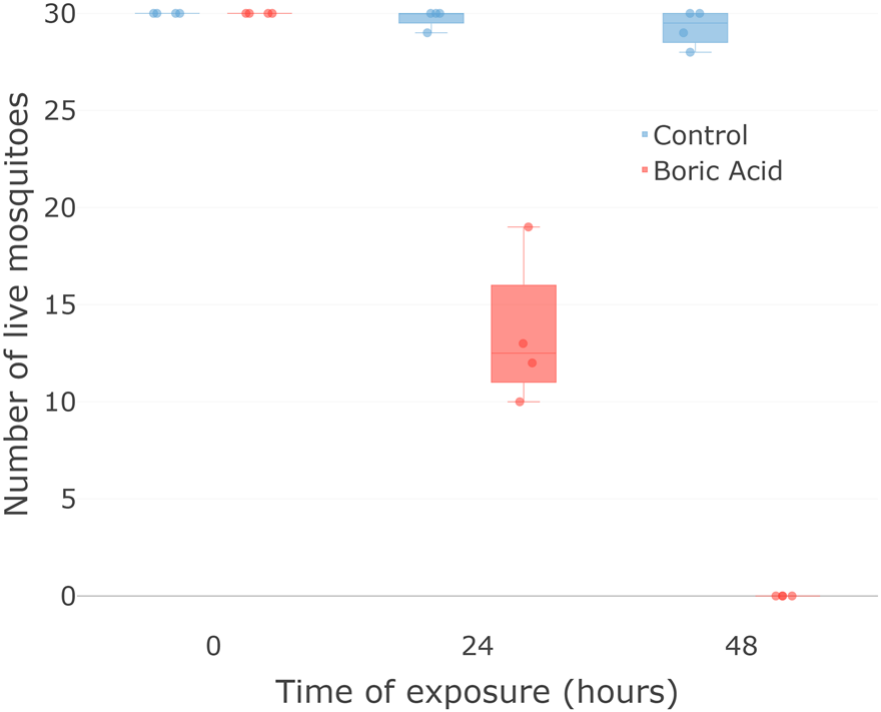
Survival assessment of mosquitoes exposed to the device. All mosquitoes (*n* = 30) exposed to toxic devices died after 48 h of exposure. When presented with non-toxic device almost all survived. Box plots indicate median 25% and 75% quartiles. Error bars indicate maximum and minimum values; each dot indicates a separate experimental replicate

We measured survival in mosquitoes exposed to toxic DABS and compared to mosquitoes exposed to control DABS in 20 × 20 × 20 cm cages in four independent replicates. An average of 13.5 (*n* = 4, SE = 1.94) out of 30 mosquitoes exposed to toxic DABS survived the first 24 h post-exposure. All mosquitoes had died by 48 h post-exposure (Fig. 2). In contrast, in the control group an average of 29.75 (*n* = 4, SE = 0.25) out of 30 mosquitoes survived 24 h post-exposure, and an average of 29.25 (*n* = 4, SE = 0.48) specimens survived 48 h post-exposure. Differences between toxic and control treatments were highly significant at 24 h (*t*_(7)_ = 8.32, *P* < 0.001) and 48 h (*t*_(7)_ = 61.1, *P* < 0.001) post-exposure.

#### Series 1.2: Characterization of the biological mode of action of the device

**Fig. 3:**
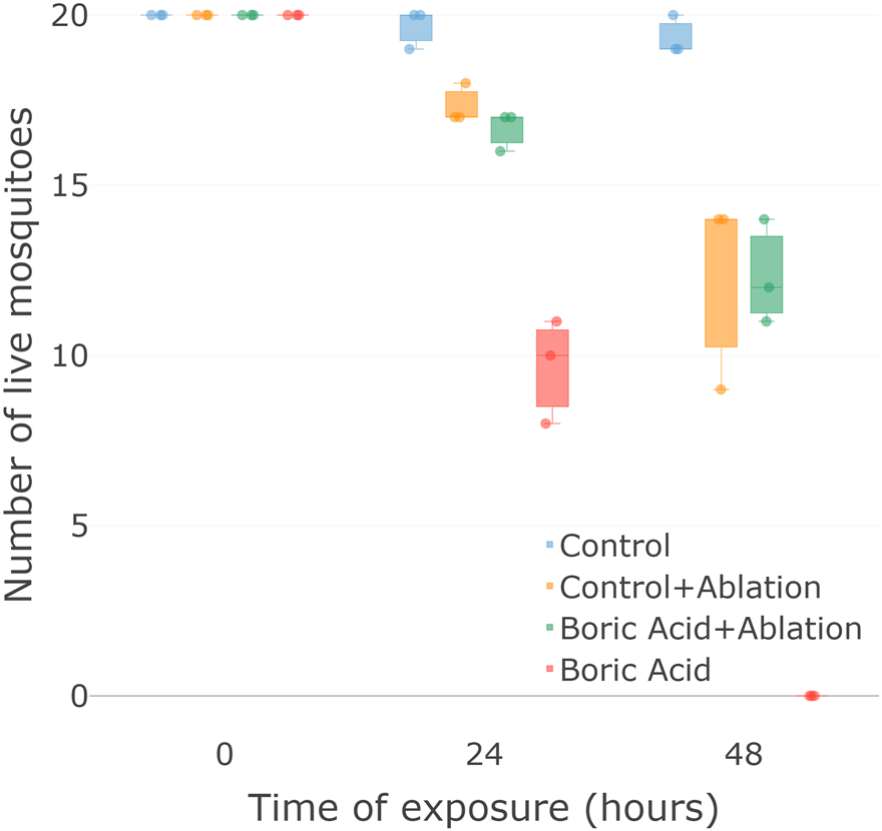
Uptake mechanism of the toxic component. Toxic effect is dependent on the ability of mosquitoes to ingest the toxic component. When mosquitoes are able to ingest the toxic component all mosquitoes (*n* = 20) died after 48 h (red). Mosquitoes with ablated mouthparts died equally regardless of the toxic or non-toxic condition of the device (green and yellow). Box plots indicate median 25% and 75% quartiles. Error bars indicate maximum and minimum values. Each dot indicates a separate experimental replicate

We disrupted the feeding parts of mosquitoes and examined survival in those exposed to toxic DABS compared to those exposed to control DABS. After 48 h, all mosquitoes that could still feed (i.e. mosquitoes with an intact proboscis) died when exposed to the toxic devices, while an average of 19.33 (*n* = 3, SE = 0.29) out of 20 survived when exposed to the non-toxic control devices. Among mosquitoes that could not feed (i.e. those with ablated proboscis), an average of 12.33 out of 20 survived whether they were exposed to toxic devices (*n* = 3, SE = 0.87) or control devices (*n* = 3, SE = 1.65). Significant differences were found between the four treatments (*F*_(3, 2)_ = 70.55, *P* < 0.001). *Post-hoc* pairwise comparisons determined that (i) the mortality of ablated mosquitoes exposed to toxic devices was not significantly different from the mortality of ablated mosquitoes exposed to control devices; and (ii) the mortality of ablated mosquitoes was significantly different from the mortality of whole mosquitoes exposed to toxic devices and whole mosquitoes exposed to control devices (Fig. 3).

**Fig. 4:**
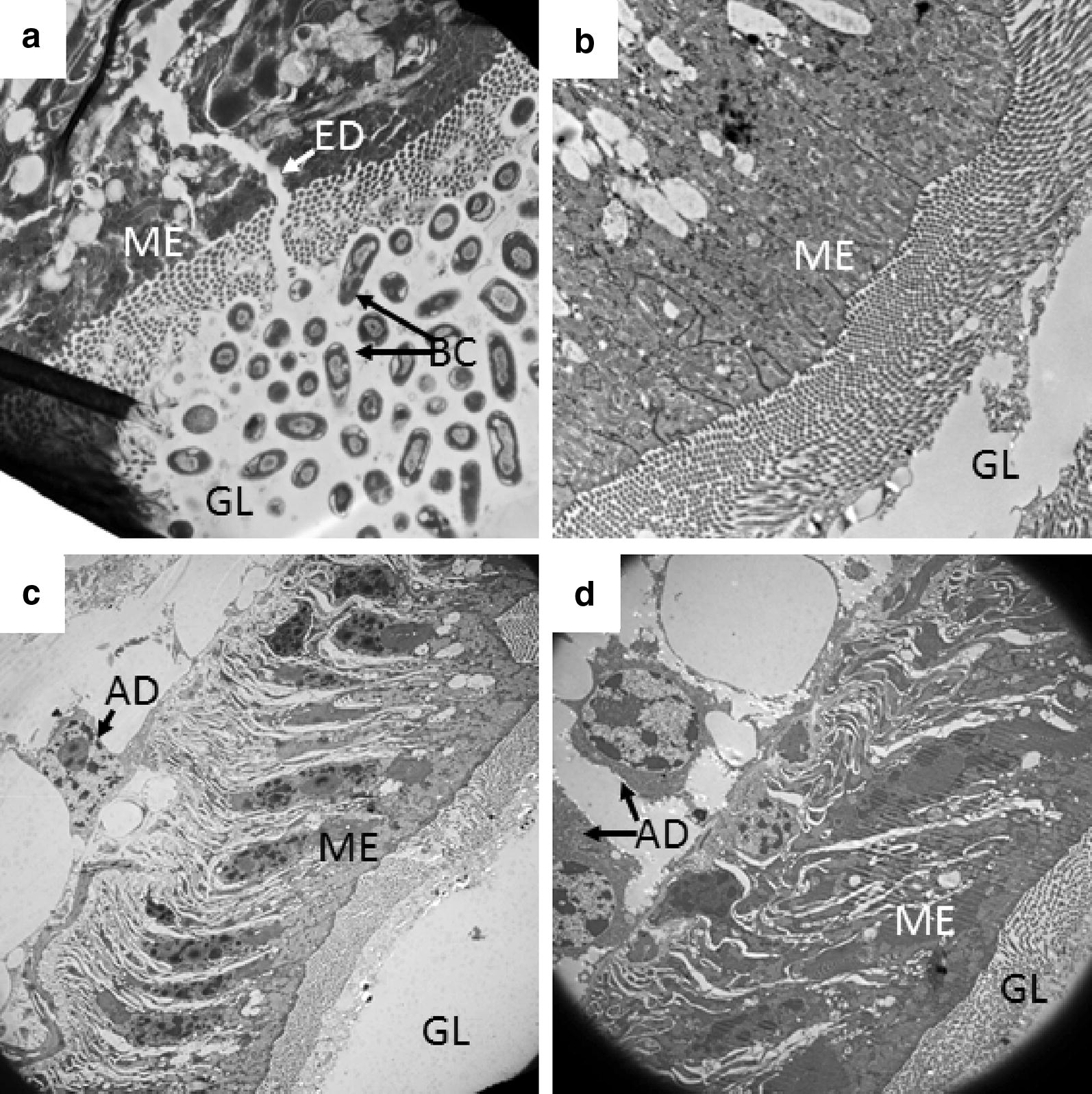
Histopathological effects on the midgut. Longitudinal sections of *Ae. aegypti* posterior midgut. **a**, **c**–**d** Mosquitoes exposed to toxic devices. Specimens exposed to toxic devices showed disruptions in the gut integrity (ED, **a**). **b** Mosquito exposed to control (i.e. non-toxic) device. Because of the even distribution of adjacent bacterial cells in the gut lumen, this disruption is unlikely to be the result of sample processing for electron microscopy. *Abbreviations*: AD, adipocyte; BC, bacterial cells in gut lumen; ED, epithelial disruption; GL, gut lumen; ME, midgut epithelium. *Magnifications*: **a**, 15,000×; **b**, 10,000×; **c**, 3000×; **d**, 5000×

Mosquitoes that had ingested toxic sugar solution presented histological abnormalities in the posterior midgut (Fig. 4). Electron micrographs revealed disruptions in the continuity of the gut epithelium (Fig. 4a), as well as abnormal-looking adipocytes in the surrounding tissue (Fig. 4c, d). Additionally, we observed an increase in both the size and number of basal infolds in the gut epithelial cells (not shown in micrographs). We hypothesize that boric acid ingestion is the cause of these pathological changes, which contributed to the mortality observed in specimens exposed to the toxic devices. Microscopic images of individuals exposed to control devices presented none of these pathologies in the posterior midgut (Fig. 4b).

#### Series 1.3: Assessment of mosquito physiological status on DABS effectiveness

We measured survival in blood-fed and parous mosquitoes exposed to both toxic and control devices. Both blood-fed and parous mosquitoes presented lower survival when exposed to toxic devices than when exposed to control devices.

Forty-eight hours after exposure to toxic devices, an average of 19.33 (*n* = 3, SE = 0.99) out of 30 blood-fed females survived. By the end of the experiment (72 h after exposure to toxic devices) an average of 2.67 (*n* = 3, SE = 1.76) out of 30 blood-fed mosquitoes survived. In contrast, 72 h after being exposed to non-toxic devices, an average of 27 (*n* = 3, SE=0.99) out of 30 blood-fed mosquitoes had survived (Additional file 2: Figure S2). Differences between control and toxic treatment survival were significant at 48 h (*t*_(5)_ = 5.75, *P* < 0.01) and 72 h (*t*_(5)_ = 12, *P* < 0.001) post-exposure.

Parous female mosquitoes showed a similar trend, with average survivals of 10.33 (*n* = 3, SE = 2.02) and 0 (*n* = 3, SE = 0) specimens following 24 and 48 h after exposure to toxic devices, respectively (Additional file 2: Figure S2b). In the non-toxic control group, an average of 29.33 specimens survived 48 h post-exposure (*n* = 3, SE = 0.33). Differences between control and toxic treatment survival curves were significant at 24 h (*t*_(5)_ = 9.25, *P* < 0.001) and 48 h (*t*_(5)_ = 87.99, *P* < 0.001) post-exposure.

#### Series 1.4: Assessment of shelf-life of the DABS device

We tested the shelf life of DABS by measuring survival of mosquitoes exposed to DABS that had been stored for different periods of time (38, 80 and 118 days), compared to those exposed to control DABS. When exposed to devices stored for 38 days, 30 out of 30 mosquitoes died at 24 hours, while an average of 28.67 (*n* = 3, SE = 0.33) mosquitoes exposed to control conditions survived 48 h post-exposure (Additional file 3: Figure S3a). Differences in survival between conditions were highly significant at 48 h post-exposure (*t*_(5)_ = 86, *P* < 0.001).

When using toxic devices stored for 80 days, an average of 5 (*n* = 3, SE = 0.58) mosquitoes survived 24 h post-exposure, and 0 mosquitoes survived 48 h post-exposure. In contrast, an average of 29.33 (*n* = 3, SE = 0.67) mosquitoes exposed to control conditions survived 48 h post-exposure (Additional file 3: Figure S3b). Differences in survival between conditions were highly significant at 48 h post-exposure (*t*_(5)_ = 44, *P* < 0.001).

On average, 28.33 (*n* = 3, SE = 0.33), 10.66 (*n* = 3, SE = 2.67), and 0 mosquitoes exposed to toxic devices stored for 118 days survived at 24 h, 48 h and 72 h post-exposure, respectively (Additional file 3: Figure S3c). Differences in survival between conditions were highly significant at 48 h (*t*_(5)_ = 6.95, *P* < 0.01) and 72 h (*t*_(5)_ = 87.99, *P* < 0.001) post-exposure.

### Semi-field experiments

We assessed the attractiveness of DABS by measuring mortality in mosquitoes exposed to DABS compared to mosquitoes not exposed to DABS in experimental houses. When exposed to DABS in semi-field trials (Series 2.1, Additional file 4: Figure S4), mosquito mortality was 0.0–6.0% (mean: 2.0%, SE: 0.9%) in the control and 17.0–57.1% (mean: 36.7%, SE: 5.3%) in the treatment house after 24 h (*t*_(5)_ = − 7.0, *P* < 0.001). At 48 h, mortality was 0.0–18.0% (mean: 5.4%, SE: 2.4%) in the control and 22.0–51.1% (mean: 38.9%, SE: 3.9%) in the treatment house (*t*_(5)_ = − 5.36, *P* < 0.01). At 72 h, mortality was 0.0–4.1% (mean: 0.7%, SE: 0.6%) in the control and 0.0–4.0% (mean: 1.4%, SE: 0.6%) in the treatment house (*t*_(5)_ = − 0.80, *P* > 0.05). The cumulative mortality of the control was 4.1–18.0% (mean: 8.2%, SE: 1.9%) and 54.0–98.0% (mean: 76.9%, SE: 6.2%) in the treatment house (*t*_(5)_ = − 8.37, *P* < 0.001). Most mosquito mortality was observed within the first 48 hours of the experiment, with no difference in mosquito mortality after this time period.

**Fig. 5:**
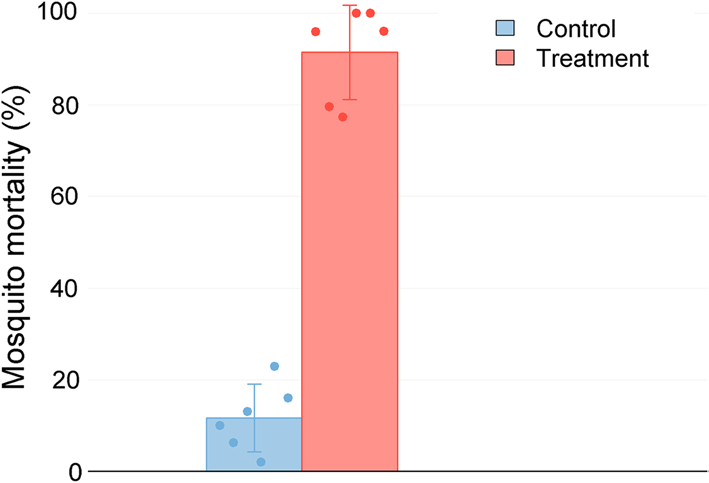
Mortality of mosquitoes when exposed to DABS for 48 h (Series 2.2). Mosquitoes were exposed to DABS for 48 h; mosquito mortality was calculated immediately after the exposure period. Mean control and experimental house mortalities are shown as bars, and standard deviation as error lines. Each dot indicates a separate experimental replicate

When exposed to DABS for 48 h (Series 2.2, Fig. 5), mosquito mortality was 2.0–22.9% (mean: 11.7%, SE: 2.8%) in the control and 77.3–100.0% (mean: 91.5%, SE: 3.8%) in the treatment house (*t*_(5)_ = − 17.0, *P* < 0.001), indicating high mortality from 48 h of exposure to DABS in the treatment houses.

When alternative attractants were included in the experimental houses (Series 2.3, Additional file 5: Figure S5), mosquito mortality was 2.0–32.7% (mean: 14.1%, SE: 4.1%) in the control and 68.0–100.0% (mean: 89.6%, SE: 4.5%) in the treatment house (*t*_(5)_ = − 12.90, *P* < 0.001), indicating that DABS results in high mortality even in the presence of a competing attractant.

When comparing the results of 24 h (Series 2.1) to 48 h of exposure (Series 2.2), 48 hours of exposure resulted in higher mortality at 48 h (*t*_(10)_ = − 8.78, *P* < 0.001) in the treatment group (Additional file 6: Table S1), with no difference in the control groups (*t*_(10)_ = − 1.55, *P* > 0.05).

When comparing 48 h of exposure to DABS only (Series 2) and 48 h of exposure to DABS in the presence of a competing attractant (Series 2.3), there was no effect of a competing attractant on the effect of DABS on mosquito mortality (*t*_(10)_ = 0.28, *P* > 0.05) in the treatment group (Additional file 6: Table S1). High mortality from 48 hours of DABS exposure was observed despite the presence of a competing attractant.

## Discussion

These experiments demonstrate that DABS can strongly impact the mortality of female *Ae. aegypti* under laboratory and semi-field conditions. In these settings, we show that mortality occurs within the first 48 hours of exposure to our devices. In addition, DABS attract and kill *Ae. aegypti* even in the presence of an alternative sugar source. To the best of our knowledge, this device is the only known “dry” ATSB. The simple and economic design lends itself to in-home use in resource-limited settings where *Ae. aegypti* target human hosts and transmit dangerous arboviruses.

Our assessment of the biological action of the devices provides an insight into the mechanism by which low concentrations of boric acid affect *Ae. aegypti*. We determined that boric acid enters the insect body by ingestion, further supporting the notion that this inorganic pesticide acts as a stomach poison, as previously suggested [32, 33]. Based on our electron microscopy analysis, we hypothesize that the ingestion of boric acid disrupts the integrity of the gut epithelium.

Considering that the proposed mechanism by which boric acid exerts its toxic effect (gut disruption) is markedly different from the neurotoxic mechanism by which most traditional pesticides cause mortality, we propose that our devices have the potential to act as efficient complementary tools to combat the spread of resistance to traditional pesticides. By combining the use of DABS with traditional pesticides in the same areas, it would be possible to target two different and crucial systems (namely, the nervous and digestive systems) in the insect body simultaneously, thereby reducing the mosquito’s probability of survival and decreasing the probability of the development of insecticide resistance.

We observed significant mortality of blood-fed female *Ae. aegypti* exposed to the DABS device, albeit at a lower rate than for starved females. Interestingly, the largest drop in survival probability in blood-fed females is observed between 48 h and 72 h post-exposure to the device (Fig. 5), suggesting that after 48 h, females have already used imbibed blood for the development of eggs and are keen to search for further meals. Based on this evidence, it is plausible to suggest that if employed in the field, DABS devices may be efficient in killing female mosquitoes of various physiological states, including females that have already ingested blood, a particularly important group for disease transmission.

Novel vector control methods have the potential to serve as critical tools in the public health effort to control persistent and emerging vector borne diseases. Various designs of ATSBs have had promising field trials for potential control of *Aedes albopictus* Skuse, 1894, *Anopheles* spp. and *Culex* spp. [15–17, 20, 21, 24]. Previous research shows that several formulations of ATSBs can achieve *Ae. aegypti* mortalities above 80% in laboratory settings [16, 25], but results from ATSBs in semi-field or field settings have been mixed. Early field trials did not show a positive effect of ATSBs on *Ae. aegypti* [26, 27]; however, a recent field trial in Bamako, Mali, showed promising success [31]. The principle barrier to field trial success appears to be the ability to attract *Ae. aegypti* to ATSBs and mixed results have been achieved when using floral-based attractants.

We hypothesize that our device attracts *Ae. aegypti* with strong visual cues (as opposed to a chemical) as an attractant. *Aedes aegypti* are container breeders [34, 35], that utilized tree holes in their natural forested habitat before adapting to life in human civilization. The DABS device has a high-contrast (black and white) 28-inch^2^ surface to simulate a refuge for *Ae. aegypti* [36]. High contrast coloring has similarly been integrated into prior trap designs and has been shown to improve capture rates of *Ae. aegypti* [37]. We believe the high-contrast coloring of DABS draws *Ae. aegypti* to land on the device.

These experiments have demonstrated the effectiveness of DABS on *Ae. aegypti* in laboratory and semi-field experimental conditions. Our approach differs from most ATSB approaches in two important ways. First, we use a device with a dried sugar solution to elicit an ingestion response while other ATSBs typically use liquid sprayed on vegetation [12, 15, 17, 26]. We hypothesize that the device is a key element in the effectiveness of DABS. Similar to other dipterans [38], *Ae. aegypti* are able to evaluate surfaces with their feet, and the “taste” of a landing surface can either lead the mosquito to feed and ingest, or reject the surface [39]. Additionally, the device provides two operational advantages over spraying liquid solutions: (i) liquid solutions are more difficult to manufacture, ship, and distribute than devices; and (ii) the device can be smaller and more easily deployed. Secondly, we use a visual rather than chemical attractant to lure *Ae. aegypti* to the device. Chemical attractants add to the cost and decrease the shelf life of any device. Previous research has questioned the ability of sugar solutions alone to attract mosquitoes [26, 33], leading to research on chemical attractant additives for ATSBs, but the use of chemical attractants in ATSBs targeting *Ae. aegypti* have been unsuccessful [26, 27]. We demonstrate that a simple black-and-white visual attractant is a sufficient motivator for female *Ae. aegypti* to land on the surface of DABS even in the presence of a competing oasis. Taken together, we hypothesize that the visual cues attract *Ae. aegypti* to land on the device, upon which the presence of the dry sugar on the device’s surface entices the insect to ingest it. When this sugar solution is mixed with boric acid, ingestion results in insect mortality.

We propose that these encouraging results justify larger field trials of DABS in open-air environments. We show that 48 hours of DABS exposure leads to high mosquito mortality when used in the laboratory and in experimental houses reminiscent of peri-urban tropical housing. Furthermore, we have established that the effectiveness of DABS for killing *Ae. aegypti* is maintained even after prolonged storage periods, a characteristic that would facilitate their use in semi-field and field conditions.

Semi-field trials are a crucial step to bring a scalable, marketable product to intra domiciliary field testing. An in-home approach is ideal for control of *Ae. aegypti*, as the vector has an extremely limited flight range, often spending its entire life within a single household [5, 35, 40]. Other research with ATSBs has shown that end-users of these products prefer to have them placed indoors [14]. The successful design and placement strategy of DABS used in our experiments indicate that the device is ideal for in-home field testing.

## Limitations

These experiments were conducted under laboratory and semi-field conditions, which can only moderately emulate real-world/field conditions. Semi-field experiments were limited to nulliparous females and we cannot be certain how DABS will affect gravid or blood-fed females or males in an open-air environment, though it should be noted that DABS were equally effective in attracting and killing blood-fed and nulliparous females under laboratory conditions. It is also unclear if DABS would impact non-target insect species, such as butterflies or other pollinators, though if DABS are limited to use inside the home, it is unlikely to affect these species. Although DABS performed well in the presence of a competing attractant (100 g of apples), it is unlikely that the attractant used in our experiments are a realistic substitute for open-air field conditions. An actual home will contain many competing attractants, including human hosts. It is difficult to know if the success of DABS in semi-field conditions will be replicated in occupied homes in the field; the number and placement of DABS may need to be modified. In addition, it is unclear how end users will react to placement of DABS in their homes, although our preliminary examinations (unpublished) suggest residents are receptive of DABS and there is evidence that residents in areas of high *Ae. aegypti* burden are willing to utilize numerous home-based mosquito control products [41].

## Conclusions

With careful design and device placement consideration, we have created a promising vector control device ready for large-scale trials to test its ability to control *Ae. aegypti* in natural conditions. We demonstrated that DABS are capable of attracting and killing female *Ae. aegypti* in experimental houses, and that 48 hours in the presence of DABS leads to high mortality among female *Ae. aegypti*. Importantly, DABS were efficient at killing female mosquitoes of diverse physiological statuses, and can attract and kill female *Ae. aegypti* even in the presence of a competing attractant.

## Supplementary information

**Additional file 1: Figure S1**. Experimental houses.

**Additional file 2: Figure S2**. Effects of the physiological status of the mosquitoes on the performance of DABS. **a** DABS performance on blood-fed mosquitoes. **b** DABS performance on parous mosquitoes. Box plots indicating median 25% and 75% quartiles. Error bars indicate maximum and minimum values. Each dot indicates an independent count of mosquitoes (y-axis) exposed to control devices (blue) or toxic devices (red) at different time points (x-axis).

**Additional file 3: Figure S3.** Shelf life of DABS. Mosquito mortality using DABS stored for 38 days (**a**), 80 days (**b**) and 118 days (**c**). Box plot indicating median 25% and 75% quartiles. Error bars indicate maximum and minimum values. Each dot indicates an independent count of mosquitoes (y-axis) exposed to control devices (blue) or toxic devices (red) at different time points (x-axis).

**Additional file 4: Figure S4.** Mortality of mosquitoes when exposed to DABS over time (Series 2.1). Mean control and experimental house mortalities are shown as bars, and standard deviation as error lines. Points indicating the mortality from each individual replicate are overlaid on each experimental condition.

**Additional file 5: Figure S5.** Mortality of mosquitoes when exposed to DABS for 48 h in the presence of alternative sugar source (Series 2.3). Mean control and experimental house mortalities are shown as bars, and standard deviation as error lines. Points indicating the mortality from each individual replicate are overlaid on each experimental condition and time point.

**Additional file 6: Table S1.** Comparison of results across semi-field experimental series.

## Data Availability

The datasets used and/or analysed during the present study are available from the corresponding author upon reasonable request.

## Abbreviations

ATSB: Attractive toxic sugar bait
DABS: Dried attractive bait stations
