# Supplementary material for: Ingested insecticide to control Aedes aegypti: developing a novel dried attractive toxic sugar bait device for intra-domiciliary control
Source: Parasit Vectors. 2020 Feb 17;13:78. doi: 10.1186/s13071-020-3930-9 (PMC7027216; doi:10.1186/s13071-020-3930-9)
Supplement: Supplementary file 1 — Additional file 1: Figure S1. Experimental houses. [file 13071_2020_3930_MOESM1_ESM.pdf]

Additional File 1: Figure S1

Experimental huts (a & b) are side-by-side on raised platforms. The interior is covered with fine mesh netting (c & d), with three windows and white canvas covering the floor. The entrance is zippered with a foyer (e). One window is fitted with a trap (f—h) to measure escape.

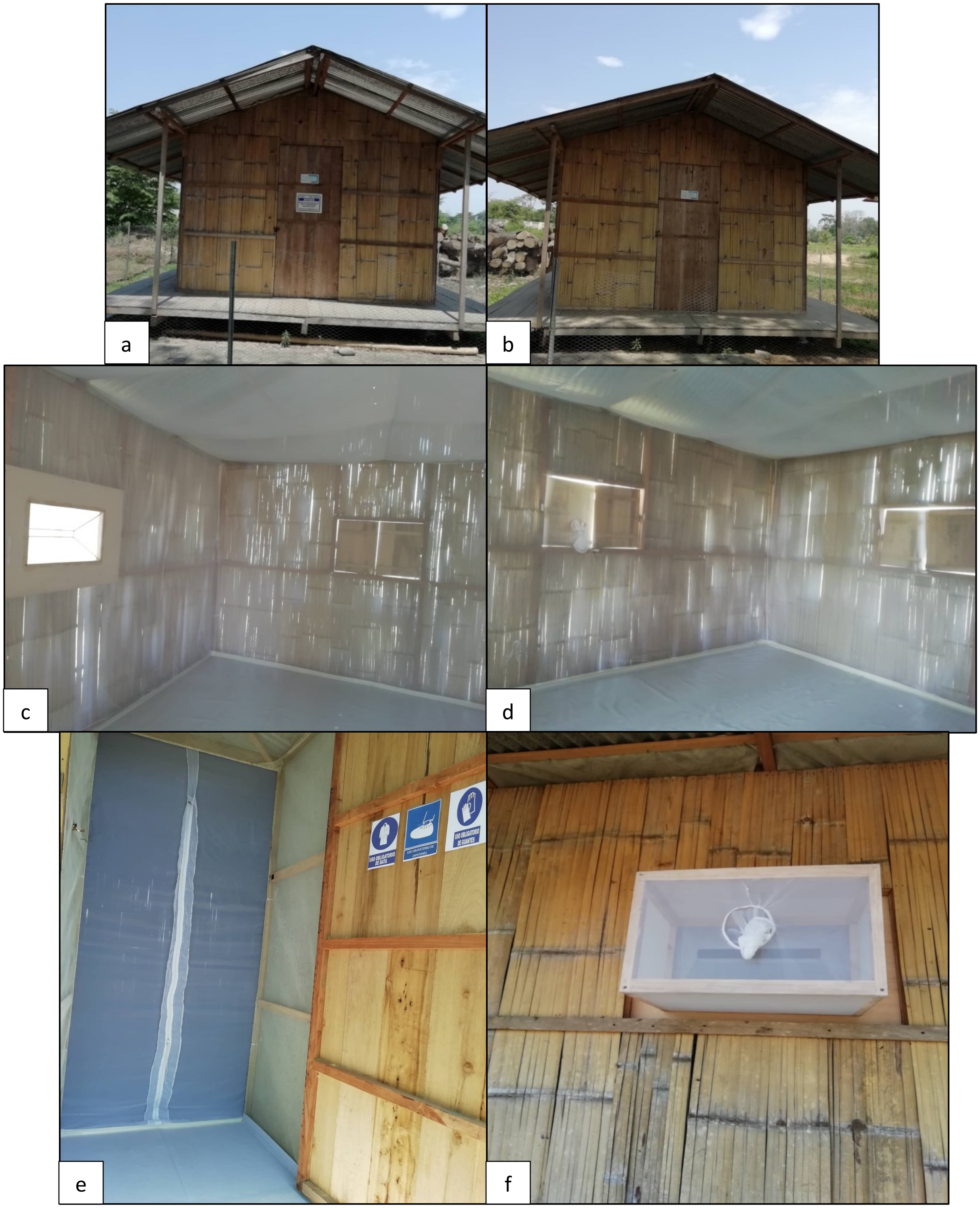

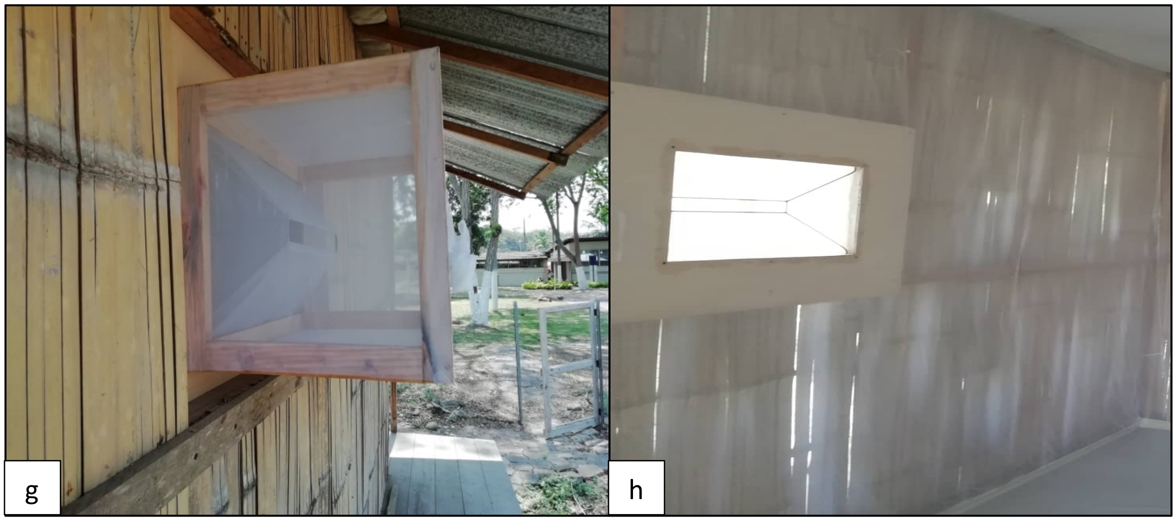
g
h
