# Supplementary figures and images for: Ingested insecticide to control Aedes aegypti: developing a novel dried attractive toxic sugar bait device for intra-domiciliary control
Source: Parasit Vectors. 2020 Feb 17;13:78. doi: 10.1186/s13071-020-3930-9 (PMC7027216; doi:10.1186/s13071-020-3930-9)

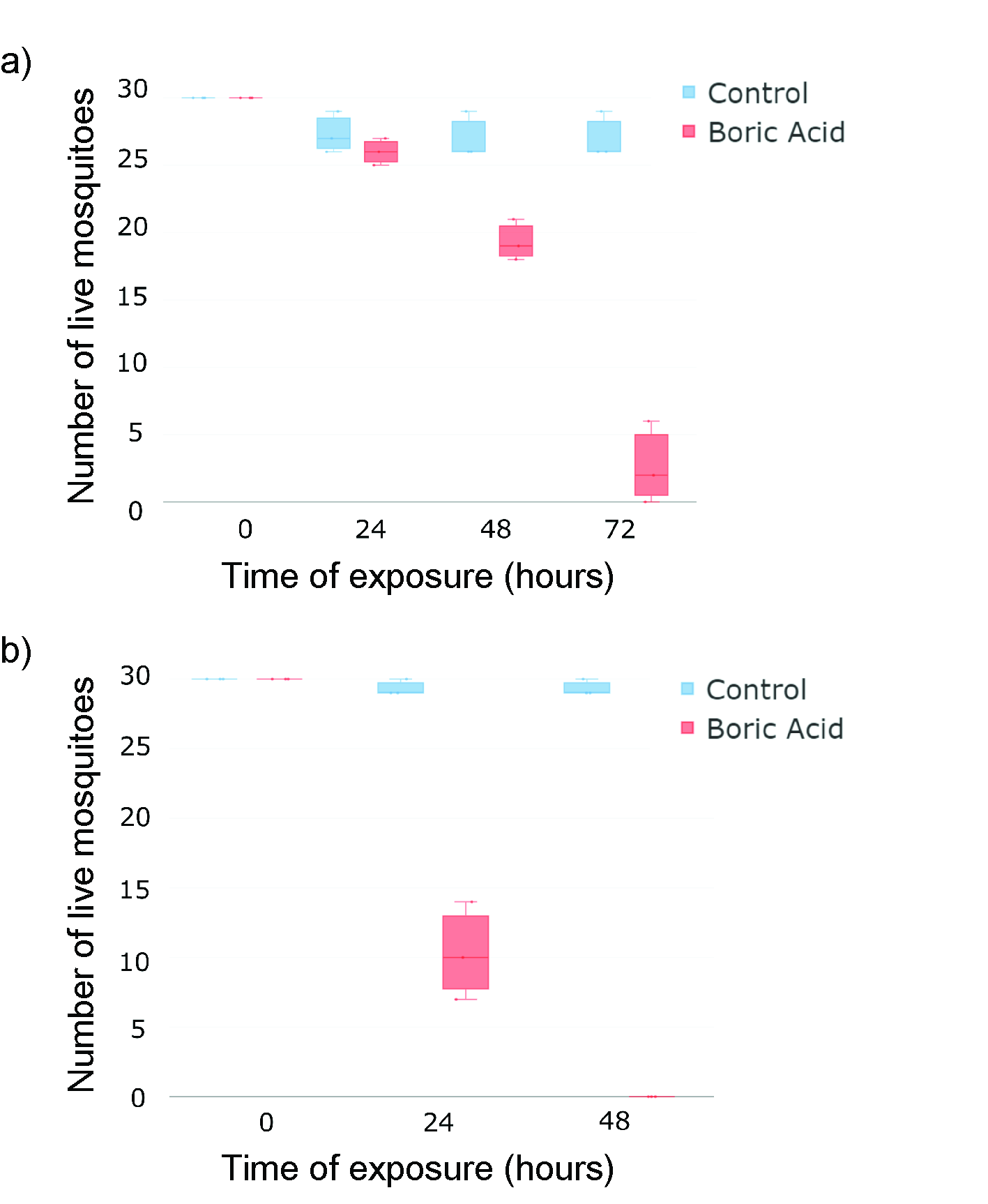

Supplement: Supplementary file 2 — Additional file 2: Figure S2. Effects of the physiological status of the mosquitoes on the performance of DABS. a DABS performance on blood-fed mosquitoes. b DABS performance on parous mosquitoes. Box plots indicating median 25% and 75% quartiles. Error bars indicate maximum and minimum values. Each dot indicates an independent count of mosquitoes (y-axis) exposed to control devices (blue) or toxic devices (red) at different time points (x-axis). [file 13071_2020_3930_MOESM2_ESM.tif]

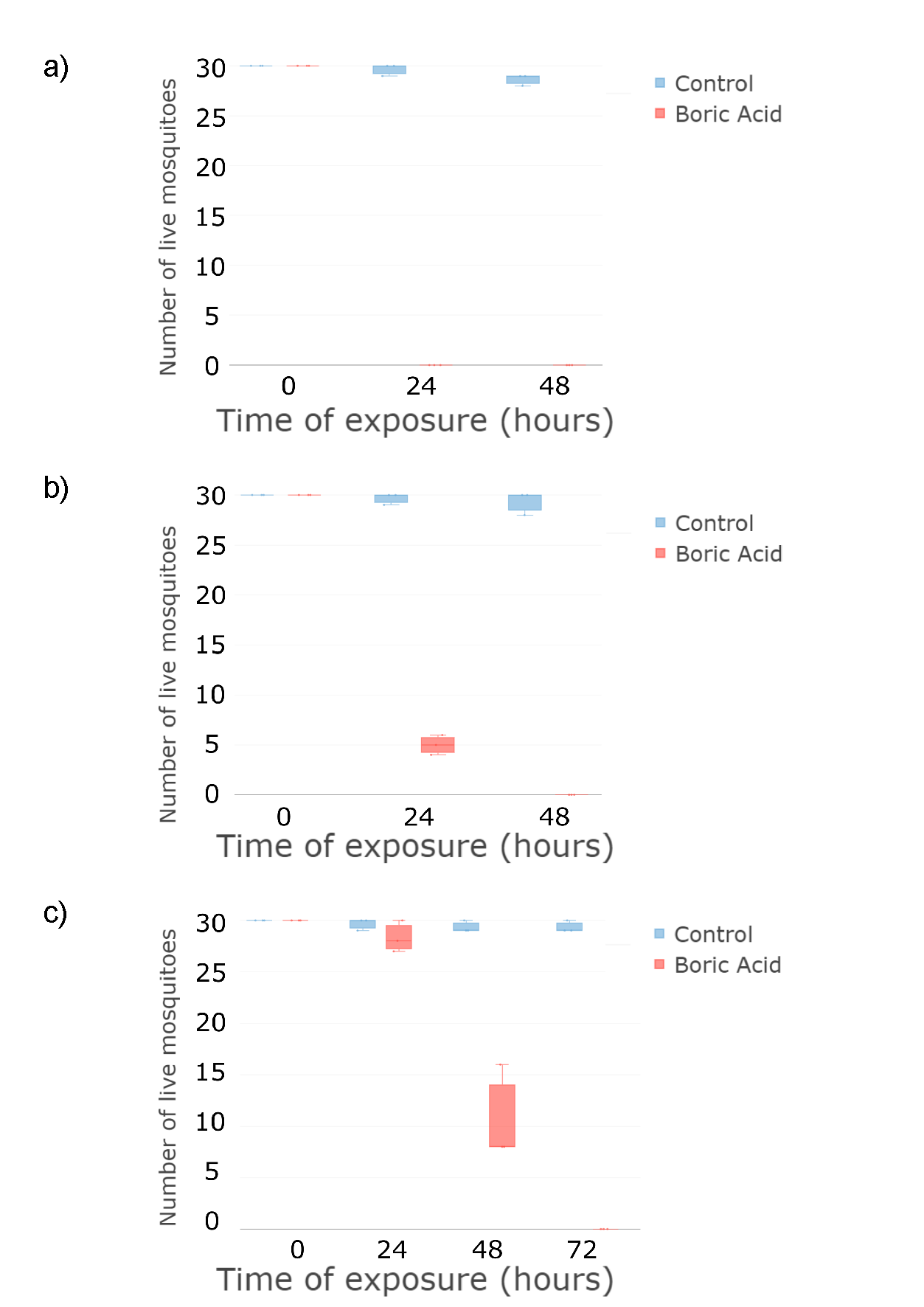

Supplement: Supplementary file 3 — Additional file 3: Figure S3. Shelf life of DABS. Mosquito mortality using DABS stored for 38 days (a), 80 days (b) and 118 days (c). Box plot indicating median 25% and 75% quartiles. Error bars indicate maximum and minimum values. Each dot indicates an independent count of mosquitoes (y-axis) exposed to control devices (blue) or toxic devices (red) at different time points (x-axis). [file 13071_2020_3930_MOESM3_ESM.tif]

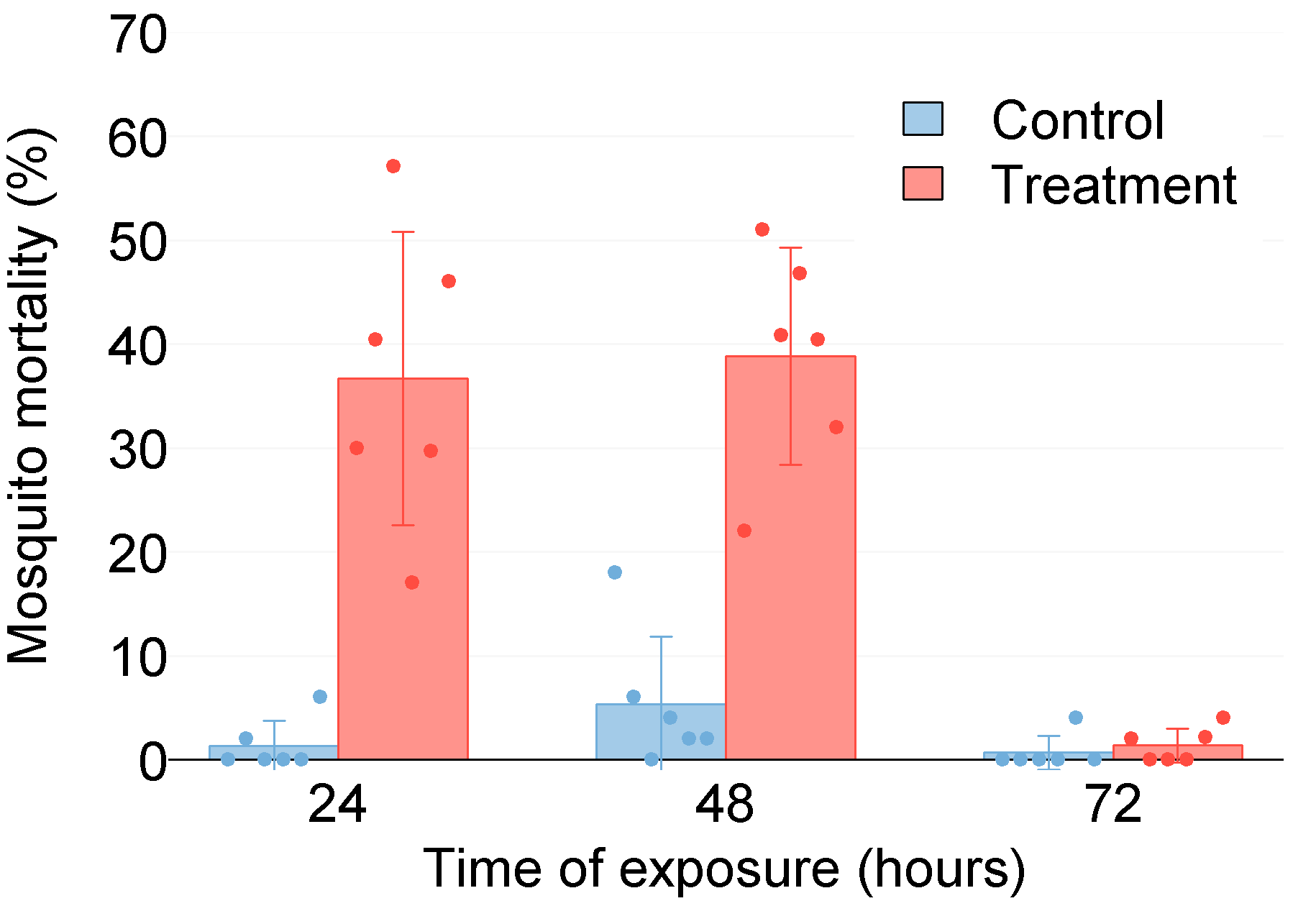

Supplement: Supplementary file 4 — Additional file 4: Figure S4. Mortality of mosquitoes when exposed to DABS over time (Series 2.1). Mean control and experimental house mortalities are shown as bars, and standard deviation as error lines. Points indicating the mortality from each individual replicate are overlaid on each experimental condition. [file 13071_2020_3930_MOESM4_ESM.tiff]

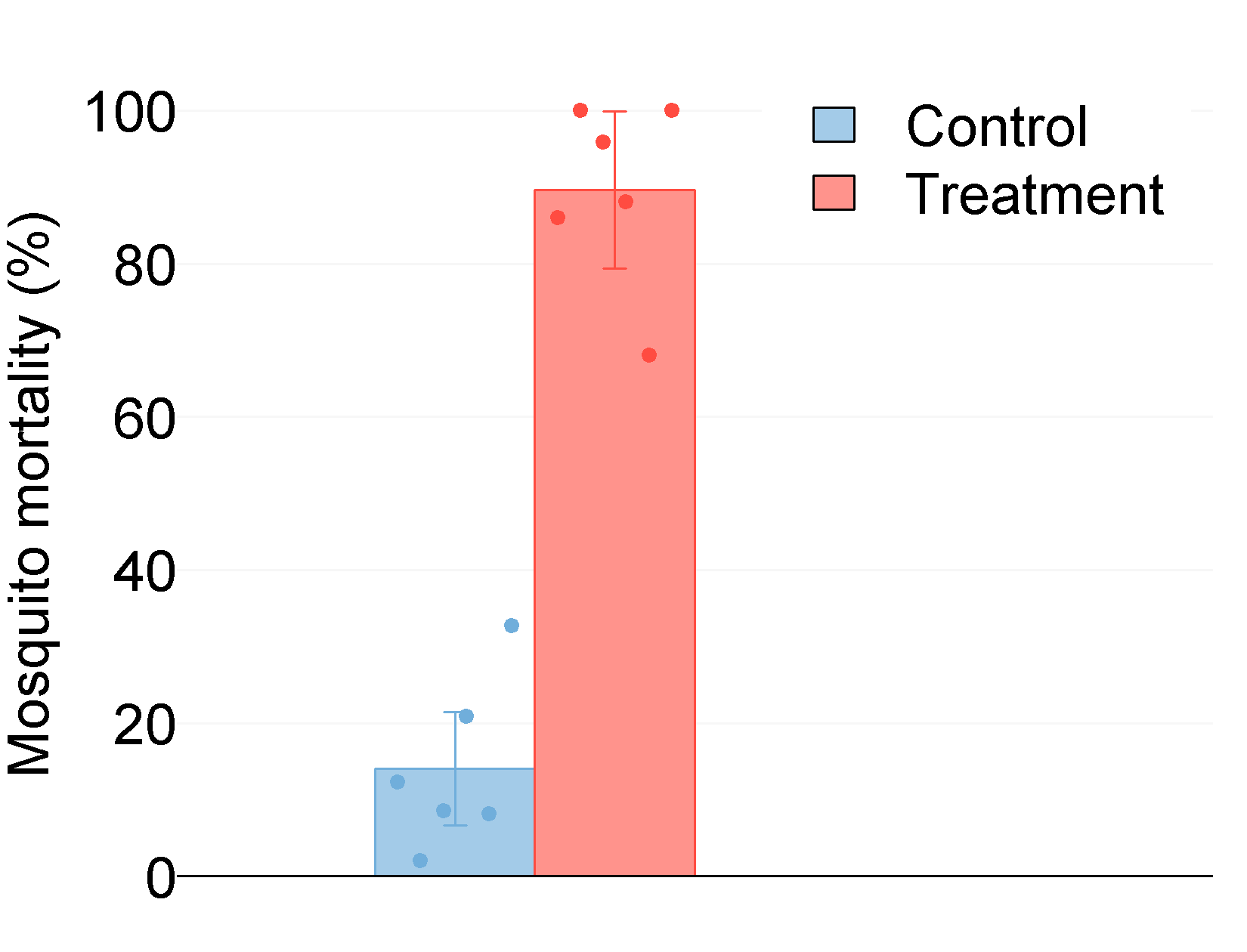

Supplement: Supplementary file 5 — Additional file 5: Figure S5. Mortality of mosquitoes when exposed to DABS for 48 h in the presence of alternative sugar source (Series 2.3). Mean control and experimental house mortalities are shown as bars, and standard deviation as error lines. Points indicating the mortality from each individual replicate are overlaid on each experimental condition and time point. [file 13071_2020_3930_MOESM5_ESM.tiff]
