# Supplementary material for: Ingested insecticide to control Aedes aegypti: developing a novel dried attractive toxic sugar bait device for intra-domiciliary control
Source: Parasit Vectors. 2020 Feb 17;13:78. doi: 10.1186/s13071-020-3930-9 (PMC7027216; doi:10.1186/s13071-020-3930-9)
Supplement: Supplementary file 6 — Additional file 6: Table S1. Comparison of results across semi-field experimental series. [file 13071_2020_3930_MOESM6_ESM.docx]

Additional file 6. **Table S1. Comparison of results across semi-field experimental series.**

| Table S1: Comparison of Results Across Semi-field Experimental Series  Mean and (standard error) of 48-hour mosquito mortality from 6-replicate series are given. Mean mortality was compared across trial series using a t-test. | | | |
| --- | --- | --- | --- |
| Experimental Conditions | | 48-hour Mortality | p-value |
| Control | 24-hour DABS exposure (Series 1) | 5.5% (2.4) | 0.151 |
|  | 48-hour DABS exposure (Series 2) | 11.7% (2.8) |  |
| Treatment | 24-hour DABS exposure (Series 1) | 38.9% (3.9) | <0.001 |
|  | 48-hour DABS exposure (Series 2) | 91.5% (3.8) |  |
| Control | 48-hour DABS exposure (Series 2) | 11.7% (2.8) | 0.671 |
|  | 48-hour DABS exposure with competing attractant (Series 3) | 14.1% (4.1) |  |
| Treatment | 48-hour DABS exposure (Series 2) | 91.5% (3.8) | 0.784 |
|  | 48-hour DABS exposure with competing attractant (Series 3) | 89.6% (4.5) |  |
